# Intraoperative Visual Field Assessment in Parietal Glioma Resection and the Role of Virtual Reality Headset-Based Mapping

**DOI:** 10.7759/cureus.84868

**Published:** 2025-05-27

**Authors:** Diego Molina-Botello, José O Santellán-Hernández, Michel Gustavo Mondragon Soto, Andrea García-Bitar, Sonia Iliana Mejía-Pérez

**Affiliations:** 1 Department of Neurosurgery, Hospital de Especialidades, Centro Médico Nacional Siglo XXI, Instituto Mexicano del Seguro Social, Mexico City, MEX; 2 Department of Neurosurgery, Instituto Nacional de Neurología y Neurocirugía Manuel Velasco Suárez, Mexico City, MEX; 3 Department of Neurosurgery, National Institute of Neurology and Neurosurgery, Mexico City, MEX

**Keywords:** glioma resection, intraoperative monitoring, parietal glioma, virtual reality headset, visual field deficit

## Abstract

The aim of this literature review is to summarize techniques used to prevent postoperative visual pathway complications during parietal glioma resection involving optic radiations, focusing on the use of virtual reality (VR) headsets during awake patient surgery.

We searched the Medline database for literature between the years 1970 and 2024. Only results that included the use of awake craniotomy for sensory mapping were included for evaluation. The search was limited to studies published in English and humans as subjects. Only studies that reported patient groups treated with either parietal glioma surgery under general anesthesia or awake conditions were included. Articles describing deep brain stimulation as the therapy were excluded because the primary focus of this literature review is on techniques such as VR and intraoperative mapping, which aim to preserve or protect the integrity of the visual pathways during glioma resection involving the optic radiations. The variables initially selected for analysis included the length of surgery, length of hospital stay, extent of resection, cost, mortality, and neurological morbidity. Cost was later removed from the studied variables because too few studies reported it. No commentaries or reviews were included.

Gliomas are the most common primary brain tumors, located mainly in the cerebral cortex. Of them, some are found in the parietal lobe, which is a convergence area for multiple stimulus integrations. After surgical resection of this lobe, patients may develop postoperative verbal, linguistic, and visual field deficits. The gold standard treatment for these tumors is surgical resection. The main technique to preserve functional pathways is by intraoperative monitoring, for which different tools have been developed in the past years.

Surgeons can test and preserve important tracts, such as the visual pathway. Intraoperative ultrasound has been shown to be an excellent, accessible, and affordable intraoperative monitoring tool. Magnetic resonance imaging-based tractography and VR-based brain mapping allow not only intraoperative monitoring, but also create a preoperative plan by demarcating the structures and margins of the tumor prior to and during surgery, increasing the success rate in maximum resection.

## Introduction and background

Parietal glioma resections carry a high risk of postoperative visual field deficits due to the proximity of the optic radiations. Preserving visual pathways while achieving maximal safe resection remains a key surgical objective. This study aims to review intraoperative techniques aimed at minimizing visual pathway injury during parietal glioma surgery, with emphasis on the use of virtual reality (VR) headsets during awake craniotomy.

A literature review was conducted using Medline and PubMed (1970-2024), focusing on studies involving intraoperative mapping of visual and sensory pathways during parietal glioma resection. Only studies in humans, in English, and excluding deep brain stimulation and reviews were included.

Conventional techniques such as MRI-based tractography, direct electrical stimulation, and intraoperative ultrasound are established tools for visual pathway preservation. Recent studies suggest VR headsets, particularly with eye-tracking capabilities, offer a novel and safe method for intraoperative visual field monitoring.

VR-based brain mapping is a promising adjunct in preserving visual function during parietal glioma resection and warrants further clinical validation.

This review was originally posted as a preprint on ResearchGate and Research Square in April 2022. Both preprints correspond to the same research and are identical to the final version presented here.

## Review

Materials and methods

For our review, we searched the Medline database for studies published between 1970 and 2024. A combination of the following MeSH terms was used for the search: craniotomy, brain neoplasms, cerebral neoplasms, supratentorial neoplasms, brain mapping, electrical stimulation, neuronavigation, time factors, focal neurological deficit, parietal glioma, VR headset, and intraoperative monitoring. In addition, an expanded PubMed search including a combination of the following terms was performed: awake craniotomy and parietal glioma surgery. Only results that included the use of awake craniotomy for sensory mapping were included for evaluation. The search was limited to studies published in English and humans as subjects.

Only studies that reported patient groups treated with either parietal glioma surgery under general anesthesia or awake conditions were included. Articles describing deep brain stimulation as the therapy were excluded. The variables initially selected for analysis included the length of surgery, length of hospital stay, extent of resection, cost, mortality, and neurological morbidity. Cost was later removed from the studied variables because too few studies reported it. No commentaries or reviews were included. A total of 37 papers were ultimately used for this review.

Gliomas and resection surgery

Gliomas

Most primary brain tumors occur in the cerebral cortex, and gliomas are the most common type of brain tumor, representing more than 70% of all brain tumors. According to their histopathology, they can be divided into astrocytoma, oligodendroglioma, and ependymoma, the last two being the most prevalent. Glioblastomas are the most frequent (65%) primary brain tumors and the most malignant histological type. At five years after diagnosis, less than 3% of glioblastoma patients survive.

In general, the three most common brain tumor locations are the frontal lobe (26%), temporal lobe (19%), and parietal lobe (12%) [1]. According to Rasmussen et al., in a 1930 patient population with diagnosis of glioblastomas (grade I-IV), 1246 of those patients had a frontotemporal glioma (65%), followed by the parietal lobe (17%), occipital lobe (9%), cerebellum (1%), and 9% in other sites [2]. Symptoms vary depending on the location; seizures are common as an initial symptom for frontal lobe tumors, deficits related to auditory processing are due to temporal lobe involvement, and speech and perception deficits are seen with parietal lobe tumors [3]. When the tumor is localized in the parietal lobe, 80% of the patients present parietal association deficit (PAD).

Surgical considerations

Despite being located at a critical junction for motor, language, visual, and sensory pathways, parietal gliomas are an underrepresented entity in the neurosurgical literature. This area represents 30% of all high-grade gliomas and 10% of low-grade gliomas. Lesions in this integrative region frequently present with subtle symptoms affecting complex neurological functions (memory, language, character, visuospatial orientation), which require specific testing performed by a neuropsychologist to be detected. These kinds of lesions, despite being localized in eloquent areas, frequently present with more subtle symptoms affecting complex neurological functions (memory, language, character, visuospatial orientation, and so on), which require specific testing performed by a neuropsychologist to be detected [4]. According to Sanai et al., there are four primary parietal areas that were identified based on clinical and radiological observation, with two additional modified subregions, resulting in six collective areas. Areas 1 to 3 of the parietal lobe are mainly visible on the superior view, where area 1 is in the supramarginal gyrus, which extends to the postcentral gyrus (area 1+), area 2 is in the superior lobe, which extends to the postcentral gyrus (area 2+), area 3 refers exclusively the angular gyrus, and area 4 comprises the cingulum [5].

The rationale of surgical treatment is based on the ability to provide an adequate specimen for histological diagnosis, genetic and molecular analysis, control the frequency of seizures (if any), diminish the burden of drugs, and improve neurological symptoms directly related to the mass effect of the tumor on the cortex or the functional fiber bundles. Finally, the extent of the resection can influence the overall survival and the period to malignant transformation (when low-grade gliomas are present) [4]. Resection is the gold standard for the treatment of gliomas, aiming for maximal resection while minimizing postoperative morbidity.

Identification of cerebral areas involved in motor, language, memory, and visuospatial functions needs to be preserved during surgery through the intraoperative use of brain mapping techniques.

Patient selection

Thorough patient evaluation and selection are required to customize surgical planning and intraoperative mapping based on anatomo-functional characteristics of the patient and the lesion. Mild deficits are usually related to direct mass effect or infiltration of the tumor on the cortex or subcortical white matter tracts.

A neuropsychological evaluation is mandatory when considering surgery for regions such as the parietal lobe to detect subtle neurological alterations and adequately implement intraoperative testing. Neuroradiological examination is composed of the usual MRI (T1-weighted, T2-weighted, fluid-attenuated inversion recovery, and gadolinium-enhanced T1-weighted) and volumetric sequences, which are useful to measure tumor volume and determine its relationship with various structures, such as major vessels. Further studies would include magnetic resonance (MR) spectroscopy, which provides information on the metabolic role of the tumor, and perfusion MRI studies, which may reveal areas of hyperperfusion related to malignant transformation.

Functional studies, such as the functional MRI (fMRI), and anatomical studies, such as diffusion tensor imaging (DTI), are useful to assess motor and language tasks and are utilized to evaluate the hemispheric dominance. DTI provides a reconstruction of the subcortical fiber bundles involved in motor (corticospinal tract), language (superior longitudinal fasciculus, inferior fronto-occipital fasciculus, inferior longitudinal fasciculus, and uncinate fasciculus), visuospatial function, and the visual tracts, which run around or inside the tumor mass. Data can be loaded into the neuronavigation system and help the surgeon in planning the resection [4,6]. Visual acuity should be evaluated, with an assessment and the Humphrey automated perimetry to register campimetric defects or homonymous hemianopia [7].

Types of craniotomies

Modern awake craniotomy originated as a means of excising epileptogenic foci and, more recently, resecting tumors in functional cortical regions. One of its main advantages includes defining tumor margins to maximize resections while preserving cortical or subcortical function. It has been associated with decreased postoperative morbidity, decreased intensive care admissions, and overall length of hospital stay. Also, it allows the surgical team to perform an intraoperative mapping of important functional brain regions [8].

In a literature review, 17 patients diagnosed with WHO grade II and III astrocytomas with no preoperative visual deficit underwent awake craniotomy, the intraoperative techniques employed included direct electrical stimulation mapping of the visual pathways during tumor resection, in which patients were instructed to report any sudden visual disturbances, such as blurriness or field loss, while stimulation was applied. Specifically, cortical and subcortical stimulation was performed using currents ranging from 2 to 5 mA, consistent with established safety thresholds. Regions that triggered visual disturbances during stimulation were identified as eloquent areas and marked as resection boundaries. Additionally, preoperative visual field testing (Humphrey visual field test) and intraoperative visual evoked potentials (VEPs) were used as adjuncts to correlate subjective patient feedback with electrophysiological monitoring. A total of 14 surgeries resulted in quadrantanopia, one in hemianopia, and two without visual deficits, with high tolerability and patient satisfaction [9]. Awake craniotomy has been used as it has been reported to be a safe and tolerable procedure that can effectively be used to preserve visual function during resection of tumors infiltrating the temporal, parietal, and occipital lobes. As mentioned before, typically, a homonymous hemianopia due to damage to the optical radiations or visual cortex is a possible consequence of tumor resection [9].

Surgical technique follows the same steps as mapping during craniotomy, as described by Kim et al. [10]. Utilizing preoperative functional tests as described before as a comparison during the procedure, the patient was instructed to note any loss of vision or blurriness during stimulation, as well as the VEPs and the Humphrey visual field test scores were noted. Previous reports described stimulation between 2 mA and 5 mA. Any area of stimulation that elicited a deficit was marked as a border for the resection [9].

On the other hand, asleep craniotomy with subcortical mapping can be used in virtue of new technological advances that can be employed along with the procedure. According to Shahar et al., both phosphene-evoked stimulation and VEP recordings are limited in their utility as warning tools to alert the surgeon of an imminent anatomical disruption of the optic radiation [7]. Subcortical mapping has been described previously as performed using a monopolar cathodal stimulator on the white matter corticospinal tracts [11].

Usually, irrigation with cold Ringer's lactate solution is not required during subcortical stimulation because stimulation-induced activity does not occur in this setting [12].

Intraoperative monitoring in parietal glioma surgery

Alternative techniques to intraoperative monitoring have emerged with the purpose of preventing and avoiding postoperative complications. MRI tractography, electrophysiological monitoring, and intraoperative ultrasound are some of the alternatives that have been proposed.

Direct Electrical Stimulation

Despite the emergence of various techniques for transoperative brain monitoring, nowadays, the technique of choice for the reduction of post-surgical neurological damage is brain mapping guided by direct electrical stimulation [13]. This procedure is performed in open craniotomies with the patient being awake and has been fundamental for procedures such as the resection of brain tumors and the localization of epileptogenic foci [14].

Intraoperative Ultrasonography

Intraoperative ultrasonography (US) is an excellent alternative for glioma patients' intraoperative monitoring. It has been effective in localizing, defining margins, and making a differential diagnosis between the tumor from a cyst or necrosis; it also helps guide the surgeon in detecting residual tumor. It is easily accessible, affordable, and can locate and support the tumor mass during resection. In addition to the possibility of having the image in real time [15].

Several studies have shown that three-dimensional ultrasound, in combination with Doppler, can be used to identify vascular structures in the affected region of the brain and visualize the ventricles [16]. These methods are an excellent alternative in resource-limited areas where other monitoring resources, such as intraoperative magnetic resonance, are unavailable [17,18].

Diffusion Tensor MRI-Based Tractography

Diffusion tensor MRI-based tractography is a noninvasive magnetic resonance technique that can delineate the brain’s white matter. It works by measuring the diffusion of water molecules. Highly cellular tissues, or those with cellular swelling, exhibit a lower diffusion coefficient. The measured quantity is the diffusivity or diffusion coefficient, a proportionality constant that relates diffusive flux to a concentration gradient [19].

Some of the many uses that DTI has are during neurosurgical planning and intraoperative neuronavigation. The latter has been shown to increase tumor resection, survival, and decrease neurologic morbidity [20]. MRI-based tractography has been used to differentiate optic radiations of neighboring tracts in healthy patients and evaluate lesions that could potentially affect the visual pathway [21]. Hajiabadi et al. performed a prospective study where 25 patients with progressive visual impairment due to suprasellar mass lesions were evaluated with the use of MRI-based tractography preoperatively, intraoperatively, and immediately after tumor resection (one week and three months after surgery). They reported that 24 out of the 25 patients with visual impairment had visual recovery, concluding that it was a tool that could help to predict visual outcome in suprasellar lesions [22].

Tractography has demonstrated its use during glioma resections near the pyramidal tract (PT), allowing, with the help of cortical and subcortical motor-evoked potentials (MEPs), maximal preservation of motor function. As Ohue et al. reported, MRI-based tractography allows establishing preoperatively the relation of the tumor with certain tracts, in this case, the PT, and the threshold intensity of the subcortical MEPs has a direct and significant correlation with the distance between the resection border and the tract on postoperative DTI [23].

Functional Magnetic Source Imaging

Functional magnetic source imaging is currently the mainstay of neuroimaging. It is used to pinpoint active brain areas. Changes in neural activity regulate the amount of local blood oxygenation, and the related variation of the magnetic field homogeneity can be identified. As a result, blood oxygenation level-dependent (BOLD) functional magnetic source imaging assesses neural activity indirectly [17,24].

Signal specificity, as well as spatial and temporal resolution, are essential aspects in evaluating the efficacy of fMRI for deriving conclusions in brain research. Signal specificity guarantees that the resulting maps reflect actual neural changes, whilst spatial and temporal resolution deny our capacity to distinguish the fundamental units of active networks and the time course of different neural events, respectively. Spatial specificity increases with magnetic field intensity and can be adjusted for a given magnetic field by employing pulse sequences that are less sensitive to signals from around and within large vessels [25].

VR-based brain mapping procedures

Surgical procedures are being adapted to new technology, which is growing faster than a few years ago. The knowledge of neuroanatomy, white matter, and the correlation of structures and function during surgery is very important to get better results for patients with complex lesions. But technology knowledge is adapted to the learning curve, and the development technology curve.

It is very important to preserve the functional structures, and in this case, the optic radiation. There is a setup where participants can virtually dissect white matter and interact with all the structures at a VR laboratory. These tools are useful now for academic purposes, training, and simulating surgeries [26], but soon they will be used routinely in the operating room [27]. The use of virtual reality headsets (VRHs) during awake craniotomy and brain mapping by direct electrical stimulation has been proven safe before by Delion et al. in a 30-patient tolerance and safety study where resection of tumors near language areas was performed [14].

According to Casanova et al., the main reason of the omission in regularly mapping other cognitive functions during tumor resections other than language and motor areas is the lack of tasks that are fully compatible with the restrictive environment of an operating room and awake brain surgery procedures, but VRH technology offers a unique opportunity to develop innovative tasks for preoperative mapping of complex cognitive functions, and a controlled environment to evaluate visual pathway damage during parietal glioma resection involving or close to the optic radiation [28]. Cassanova et al. demonstrated that the use of VRH equipped with eye-tracking was useful to evaluate patients' visual fields of the VRH directly, thus giving an area of opportunity to use this kind of device in parietal glioma resection, where protecting the optic radiation is the main goal [28].

Recent literature has reinforced the value of advanced intraoperative tools in optimizing outcomes for patients undergoing parietal glioma resection. In a prospective study of 39 patients with gliomas near the optic radiations, Soumpasis et al. (2023) demonstrated that intraoperative neuromonitoring of the visual pathway using VEPs and DTI tractography was significantly associated with better visual outcomes postoperatively, highlighting the relevance of electrophysiological and tractographic metrics during surgery [29]. Meanwhile, Hellum et al. (2022) evaluated neurosurgical planning tasks using VRHs, establishing that human surgeons found VR platforms highly usable for annotation, preoperative simulation, and spatial orientation, setting the stage for broader clinical integration [30].

The parietal lobe is a convergence area of multiple stimulus integration, including verbal, linguistic, and even visual stimuli. A parietal association deficit could be defined as a lack of integration of these stimuli. The rest of the results are shown in Table 1 [31].

**Table 1:** Prevalence of cognitive and neurological complications after glioma resection. This table shows the prevalence of various cognitive and neurological complications observed in patients following glioma surgery.

Complication	Prevalence (%)	Description
Parietal association deficits (PAD)	80	Impairments of the ability to integrate multisensory information necessary for spatial orientation, body schema, attention, and higher-order cognitive functions.
Apraxia	47	A motor disorder impacting the ability to perform learned, purposeful movements.
Anomic ataxia	39.5	A condition involving coordination and motor control issues, often with cognitive dysfunction.
Clinical manifestations of Gerstmann syndrome	34.2	Symptoms such as agraphia, acalculia, finger agnosia, and left-right disorientation, often linked to parietal lobe lesions.
Visual pathway alterations	15.8	Disturbances in the visual field or higher-level visual processing, often related to tumor location or surgical impact on optic pathways.

Complications

Surgery is the gold standard for treating parietal gliomas, nevertheless, the parietal lobe has somatosensory structures and language areas, which make surgeries a great challenge [29]. The complications that may develop are basically determined by three specific factors: the tumor’s exact location, diameter, and volume. The location must be identified, assessing whether it is in the dominant or not-dominant hemisphere, the involvement of the superior or inferior parietal lobe or both, the involvement of the postcentral gyrus, and the depth [25,32]. The aggressive resection of the tumor volume renders the tumor’s remnant fragments more sensitive to subsequent therapeutic alternatives, such as radiotherapy and chemotherapy [33]. There is multiple evidence that extensive glioma resection increases survival and decreases tumor progression [34-36].

Amongst neurological complications, the most frequent alterations found in the immediate postoperative period are loss of the visual field, agraphesthesia, hemiparesis, acalculia, and astereognosis [32]. According to Russell et al., after resection of parietal lobe gliomas, neurological deterioration and improvement occur [33]. The deficits of the parietal lobe association, primarily the components of Gerstmann syndrome, are mostly related to large tumors that include the superior and inferior parietal lobules. Following resection of lesions in the nondominant hemisphere, no patient developed new hemineglect or sensory extinction. Regardless of the hemispheric dominance, patients experienced the main primary parietal lobe deficit, such as cortical sensory syndrome and visual field loss [32]. Vallar et al., with the use of direct electrical stimulation during glioma resection surgery in a case series of seven patients, described that the posterior superior parietal lobe (Brodmann area 7) is involved in the orientation of spatial attention (corresponding to the first branch of the superior longitudinal fascicle), thus its involvement in gliomas or its damage during surgery may present with a visuospatial neglect [37].

## Conclusions

Parietal lobe glioma resection is complex due to the risk of visual deficits, but techniques like MRI-based tractography, ultrasound, and VR mapping help minimize complications. These tools enhance tumor delineation and intraoperative monitoring, improving resection outcomes and reducing postoperative deficits.
